# The use of high-frequency ultrasound imaging and biofluorescence for *in vivo* evaluation of gene therapy vectors

**DOI:** 10.1186/1471-2342-13-35

**Published:** 2013-11-12

**Authors:** Nicola Ingram, Stuart A Macnab, Gemma Marston, Nigel Scott, Ian M Carr, Alexander F Markham, Adrian Whitehouse, P Louise Coletta

**Affiliations:** 1 School of Medicine, University of Leeds Brenner Building, St James’s University Hospital, Leeds LS9 7TF, UK; 2 School of Molecular and Cellular Biology, Faculty of Biological Sciences and Astbury Centre for Structural Molecular Biology, University of Leeds, Leeds LS2 9JT, UK; 3 Department of Histopathology, Bexley Wing, St James’s University Hospital, Leeds LS9 7TF, UK

**Keywords:** Biofluorescence, Ultrasound, Gene therapy, Imaging, Multi-modal, Colorectal cancer

## Abstract

**Background:**

Non-invasive imaging of the biodistribution of novel therapeutics including gene therapy vectors in animal models is essential.

**Methods:**

This study assessed the utility of high-frequency ultrasound (HF-US) combined with biofluoresence imaging (BFI) to determine the longitudinal impact of a Herpesvirus saimiri amplicon on human colorectal cancer xenograft growth.

**Results:**

HF-US imaging of xenografts resulted in an accurate and informative xenograft volume in a longitudinal study. The volumes correlated better with final *ex vivo* volume than mechanical callipers (R^2^ = 0.7993, p = 0.0002 vs. R^2^ = 0.7867, p = 0.0014). HF-US showed that the amplicon caused lobe formation. BFI demonstrated retention and expression of the amplicon in the xenografts and quantitation of the fluorescence levels also correlated with tumour volumes.

**Conclusions:**

The use of multi-modal imaging provided useful and enhanced insights into the behaviour of gene therapy vectors *in vivo* in real-time. These relatively inexpensive technologies are easy to incorporate into pre-clinical studies.

## Background

The use of non-invasive and accurate methods to determine tumour volume, as well as biodistribution and transduction imaging of novel therapeutics, is essential in experimental models *in vivo*. In particular, for gene therapy studies, knowledge of maintenance, expression and efficacy of the vector is a fundamental part of the testing process [1]. However, this is rarely achieved during the *in vivo* study of a novel gene therapy strategy, as often only longitudinal calliper measurements of xenograft growth or final histology after treatment are carried out. The spread or loss of a vector is rarely detected during the course of the experiment and for cancer treatment, not all therapies will result in a reduction in tumour volume. Therefore it is important to be able to examine the impact of a gene therapy vector during the *in vivo* testing phase using different assessment criteria, whilst being mindful of adhering to the principles of reduction, refinement and replacement in animal experiments.

Ultrasound is a non-invasive method that has been utilised recently for tumour growth studies *in vivo* and is used in the clinic for staging colorectal cancer among others [2,3]. High-frequency ultrasound (HF-US) machines are available for small animal imaging. They are relatively easy to use and give high resolution greyscale images of mouse anatomy [4]. They also give functional information on the vascular structure of xenografts through the use of contrast agents and are relatively inexpensive and portable compared to MRI machines [5]. Mechanical callipers, however, are still utilised extensively for therapeutic agent testing, especially in gene therapy applications on xenografts [6]. These are very cheap, non-invasive and allow multiple repeated measurements with no anaesthetic required. However, mechanical callipers assume that the growth of xenografts is always ellipsoid and can only measure growth above the skin surface of the animal. In addition, calliper measurements are also affected by skin thickness, subcutaneous fat layer thickness and compressibility of the tumour [7]. From our experience of xenograft growth in gene therapy and other therapeutic studies, we know that this ellipsoid growth pattern is rarely observed, especially as the tumour volume becomes large (above approximately 300mm^3^).

A gene encoding a fluorescent or luminescent protein is often incorporated into gene therapy vectors in order to enumerate transduction efficiencies *in vitro*[8,9]. Moreover, these markers are also very useful for *in vivo* studies. Optical imaging chambers can be used to image the biodistribution of a vector when administered and can give an indication of the transduction efficiency in the target cells [10]. Optical imaging systems also allow the maintenance of a vector to be determined throughout the course of treatment, as well as examining the genetic stability of the vector over time. The first paper to prove that optical imaging could be used to measure tumour growth used bioluminescence of tumour cells in rat brain and was compared to MRI scans for tumour volume [11]. Imaging of stably-transfected cell lines containing red or green fluorescent protein (RFP or GFP) has been used to measure tumour and metastatic growth [12,13]. Recent work has also shown that fluorescent intensity correlates better with tumour volume than fluorescent area [14].

In the study described herein, we aimed to determine whether the use of HF-US measurements were more accurate than mechanical callipers in assessing xenograft volumes of tumour cells which were infected before injection with an experimental gene therapy vector. The use of HF-US to provide anatomical information on tumour growth and BFI to monitor expression of a gene therapy vector in longitudinal studies, were also analysed. The vector we used was a Herpesvirus saimiri (HVS) amplicon which contains the minimal elements for episomal maintenance without infectious capabilities [9,15]. This gamma-2 Herpesvirus amplicon can incorporate large amounts of heterologous DNA using a HVS-BAC (bacterial artificial chromosome) system and infects a broad range of human cells. The amplicon was previously stably transfected into the SW480 colorectal cancer cell line and contains a constitutively active GFP gene [16]. The presence of the GFP gene enabled monitoring of its persistence during xenograft growth in this study.

## Methods

### Tumour model

The colorectal cancer cell line, HCT116 was stably-transfected with an episomally-maintained Herpesvirus saimiri amplicon incorporating the GFP gene under the control of the Cytomegalovirus (CMV) promoter. These cells were grown in Dulbecco’s Modified Eagle Medium (DMEM, Invitrogen) supplemented with 10% (v/v) foetal calf serum, and 4ul/ml Hygromycin B (Sigma, Poole U.K.) in 5% CO_2_ at 37^o^C until there were enough cells for xenograft set up (approximately 3-4 weeks from infection). Parental cell lines were grown in DMEM and serum but no Hygromycin B. Two days before injection the amplicon-transfected cells were transferred to medium without any Hygromycin B.

1 × 10^6^ each of the parental and amplicon-containing cells were collected in 100ul of serum-free DMEM and injected subcutaneously into the right flank of 8-10 week old female CD1 nude mice to form xenografts. 6 mice per group were used. All experiments were performed following local ethical approval and in accordance with the Home Office Animal Scientific Procedures Act 1986.

### Tumour volume measurement with mechanical callipers

Tumours were measured with mechanical callipers three times per week once the tumour became palpable (approximately 7-10 days following injection). Tumour volume was calculated as follows, unless otherwise stated: [17]

$$\begin{array}{l} \mathrm{Tumor}\;\mathrm{volume}=1/2\left(\mathrm{greatest}\;\mathrm{longitudinal}\;\mathrm{diameter}\right)\\ \;\left(\times \mathrm{greatest}\;\mathrm{transverse}\;\mathrm{diamete}r^{2}\right) \end{array}$$

After 40 days a final calliper measurement was taken, the xenografts were excised and weighed. If tumours exceeded the maximum permitted size of 17mm diameter, the mice were sacrificed earlier. Mechanical calliper measurements were then taken in three dimensions *ex vivo* and the following tumour volume was calculated, unless otherwise stated:

$$\mathrm{Tumor}\;\mathrm{volume}\;=\mathrm{length}\times \mathrm{height}\times \left(\pi /6\right)$$

### Anatomical imaging and tumour volume measurement using HF-US

Once per week, mice were anaesthetised using 3% (v/v) isofluorane and xenografts were imaged using a Vevo 770 high-frequency ultrasound machine (FUJIFILM VisualSonics, Inc, Toronto, Canada) equipped with a 40 MHz transducer. The focal depth of the transducer was placed at the mid-point of the centre of the tumour whilst scanning. A 3D scan of the tumour was then performed using the minimum step size possible for the length of tumour and regions of interest were drawn around the xenograft at approximately every 5 frames by an operator with extensive experience of HF-US and analysis [4]. A tumour volume was then calculated using the Vevo 770 version 3 software by creating a 3D reconstruction of these xenografts.

### Measurement of biofluorescence

Before sacrifice at day 40, xenografts were imaged in an IVIS Spectrum (PerkinElmer, Inc, Massachusetts, USA). Standard settings for GFP were used (excitation 500nm and emission detected at 540nm) in epi-illumination at high intensity. Binning was set at 8, field of view was 13.1cm and f stop was 2. Regions of interest of the same size were drawn around each xenograft and the total radiant efficiency ([photons/s]/[μW/cm^2^]) was calculated within this using Living Image version 4.2 software(PerkinElmer, Inc, Massachusetts, USA).

### Histology and morphology of xenografts

Once the xenographs were excised, photographs were taken of the intact tumours. The tumours were then cut in half and fixed in 4% (w/v) paraformaldehyde in PBS overnight. After processing and embedding in wax, sections were dewaxed, rehydrated and stained with haematoxylin and eosin. Sections were assessed by an experienced histopathologist.

### Statistical analysis

Analysis of the tumour volumes and vector expression obtained by these methods used Pearson correlations. Positive correlations produced a positive R^2^ value and were considered significant if p < 0.05. Agreement between the methods was then further analysed by Bland-Altman plots where the central line (mean of differences or bias) and 2 standard deviation (SD) limits of agreement were generated. The bias was considered significant if 0 was not included within these standard deviation lines. These calculations were carried out using GraphPad Prism version 5 (GraphPad Software, Inc, La Jolla, California, USA).

## Results

### Comparison of tumour growth curves generated using mechanical callipers or HF-US

**Figure 1:**
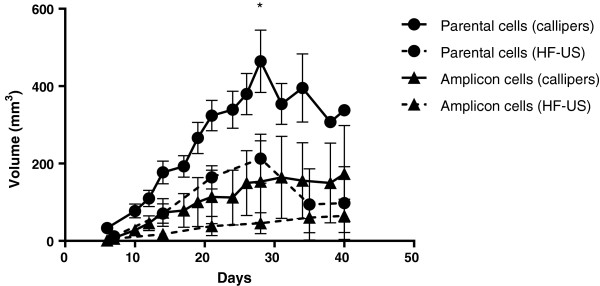
**Longitudinal growth of xenograft tumours using mechanical callipers and HF-US.** Growth of xenografts generated from each line using mechanical Vernier callipers on the external surface of the animal (*in vivo* calliper volume – solid lines) and using 3D high-frequency ultrasound scans and calculating volumes by drawing regions of interest on each frame (dotted lines). Mean volume +/- standard deviation of each group is shown (n = 5 for calliper measurements and 6 for HF-US) * denotes that mice were culled in this group after this point due to large tumour volumes (n = 2 from day 28).

HF-US was used to determine the tumour volume during the growth course of the xenografts derived from the parental cell line and amplicon-infected cell line and compared to the volume calculated from mechanical calliper measurements. The tumour volumes generated from the two methods are shown in Figure 1. The amplicon-infected xenograft tumours grew more slowly than the parental cells and this was detected by both measurement methods. Tumour volumes by HF-US generated smaller calculated tumour volumes than those using mechanical callipers. At day 28 for example, calliper assessed xenograft tumour volumes were calculated to be more than twice the volumes generated using HF-US imaging. This difference was even greater for the amplicon-infected xenografts as these were 3.3 times larger when measured using mechanical callipers compared to HF-US.

### Comparison of tumour volume measurement methods to the volume calculated using *ex vivo* calliper measurements

**Figure 2:**
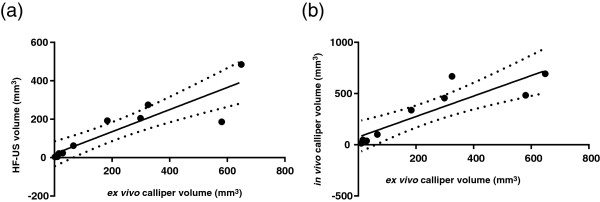
**HF-US correlates more closely to the *ex vivo *tumour volume than using mechanical callipers *in vivo*.** The tumour volume generated by HF-US correlates more closely with the final *ex vivo* calliper volume than the *in vivo* calliper volume. **(a)** The Pearson correlation plot of HF-US volumes versus *ex vivo* volumes has a higher R^2^ value (R^2^ = 0.7993, 95% CI = 0.6342-0.9724,p = 0.0002, two-tailed) than *in vivo* calliper volumes versus *ex vivo* volumes **(b)** (R^2^ = 0.7867, 95% CI = 0.5421-0.9761, p = 0.0014, two-tailed). The solid line denotes line of best fit and dotted lines indicate the 95% confidence band, n = 10.

HF-US measurements correlated more closely than mechanical callipers (denoted as *in vivo* callipers on the graphs) to the final *ex vivo* calliper measurement at the end of the period of xenograft growth which is our most accurate measurement (Figure 2 a and b). Thus the tumour growth curves in Figure 1 are an over-estimation if mechanical callipers are used compared to HF-US measurements. Alternative formulae for tumour volume calculation for both *in vivo* and *ex vivo* calliper measurements were examined and compared to HF-US (Table 1) [17]. As before, HF-US measurements correlated more closely to either *ex vivo* volume formula than any *in vivo* volume formula and no difference in correlation was found between the two *ex vivo* volume formulae and HF-US volumes. Using the formula π/6 × (L × W)^3/2^ for *in vivo* calliper volumes gave a higher correlation to both HF-US volumes and to mass of tumour than the other two equations.

**Table 1:** Pearson correlation coefficients of xenograft tumour volumes using different ellipsoid formulae and measured using mechanical callipers, HF-US or mass

		**π/6 × L × W × H**	**0.5 × L × W × H**	**HF-US**	**Mass (g)**
0.5 × L × W^2^	**R**^**2**^	**0.7867**	**0.7867**	**0.8576**	**0.7843**
	**95% CI**	**0.5421-0.9761**	**0.5421-0.9761**	**0.7110-0.9827**	**0.4811-0.9792**
	**p**	**0.0014**	**0.0014**	**0.0001**	**0.0034**
π/6 × L × W^2^	**R**^**2**^	**0.7867**	**0.7867**	**0.8576**	**0.7843**
	**95% CI**	**0.5421-0.9761**	**0.5421-0.9761**	**0.7110-0.9827**	**0.4811-0.9792**
	**p**	**0.0014**	**0.0014**	**0.0001**	**0.0034**
π/6 × (L × W)^3/2^	**R**^**2**^	**0.8325**	**0.8325**	**0.8636**	**0.8492**
	**95% CI**	**0.6300-0.9817**	**0.6300-0.9817**	**0.7223-0.9835**	**0.6184-0.9860**
	**p**	**0.0006**	**0.0006**	**0.0001**	**0.0011**
HF-US	**R**^**2**^	**0.7993**	**0.7993**		**0.8470**
	**95% CI**	**0.6342-0.9724**	**0.6342-0.9724**		**0.6135-0.9857**
	**p**	**0.0002**	**0.0002**		**0.0012**
Mass (g)	**R**^**2**^	**0.9254**	**0.9254**		
	**95% CI**	**0.7580-0.9946**	**0.7580-0.9946**		
	**p**	**0.0005**	**0.0005**		

Three different formulae for generating *in vivo* tumour volumes using callipers are shown to the left and two different formulae for *ex vivo* tumour volumes using callipers on top. Each have been subject to pairwise comparison to determine the different correlation coefficients.

### Comparison of tumour volume measurement methods to final tumour mass

**Figure 3:**
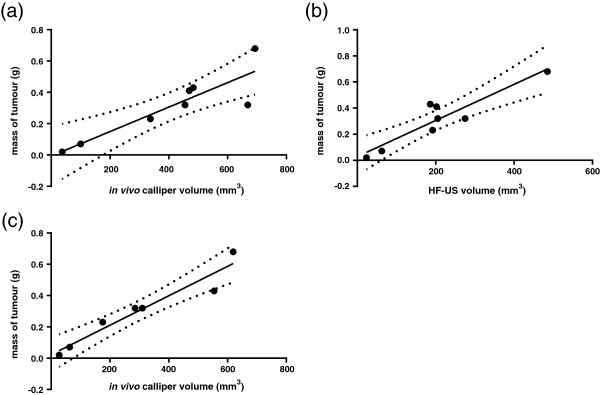
***Ex vivo *callipers and HF-US correlated well to tumour mass.** Final tumour mass correlated most strongly with *ex vivo* calliper volume. Pearson correlations showed that *in vivo* calliper volumes correlated the least with tumour mass (Figure 3**a**, R^2^ = 0.7843, 95% CI = 0.4811-0.9792, p = 0.0034), followed by HF-US volume (Figure 3**b**, R^2^ = 0.8470, 95% CI = 0.6135-0.9857, p = 0.0012) whereas *ex vivo* calliper volume showed the best correlation (Figure 3**c**, R^2^ = 0.9254, 95% CI = 0.7580-0.9946, p = 0.0005). Smaller tumours were not accurately weighed by the balance therefore n = 8.

**Figure 4:**
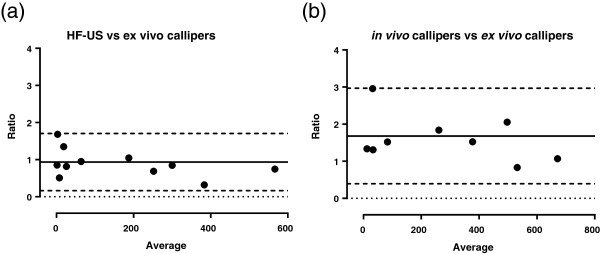
**Bias assessment of each method for tumour volume calculation.** HF-US volumes show less bias than *in vivo* calliper volumes when compared to *ex vivo* calliper volumes. The ratios of HF-US to *ex vivo* calliper volumes (y-axis) were compared to the average value of the measurements (x-axis). Bland-Altman plots were generated comparing the bias between HF-US and *ex vivo* calliper volumes **(a)** and *in vivo* calliper volumes compared to *ex vivo* calliper volumes **(b)**. The solid line denotes the bias (the average of the differences between the two measurement methods) and the dashed lines define the 95% confidence limits. The dotted line defines zero. HF-US could detect much smaller tumour volumes than callipers therefore n = 11 in **(a)** and n = 10 in **(b)**.

After sacrifice, the resulting xenograft tumours were excised and weighed. Using Pearson correlation coefficients and linear regression analysis, final *in vivo* calliper measurements had a lower correlation coefficient to tumour mass than HF-US. The tumour volumes calculated from *ex vivo* calliper measurements of the excised xenograft had the highest correlation coefficient to tumour mass (Figure 3 a, b and c and Table 1). Bland-Altman graphs show a smaller 95% confidence interval between HF-US volumes and the *ex vivo* calliper measurement compared to the confidence interval between final *in vivo* calliper and the *ex vivo* calliper measurements (Figure 4a and b). This demonstrates a smaller difference between HF-US and the *ex vivo* calliper measurement methods than between *in vivo* and *ex vivo* callipers.

### HF-US imaging and BFI of tumour anatomy and gene therapy vector expression

**Figure 5:**
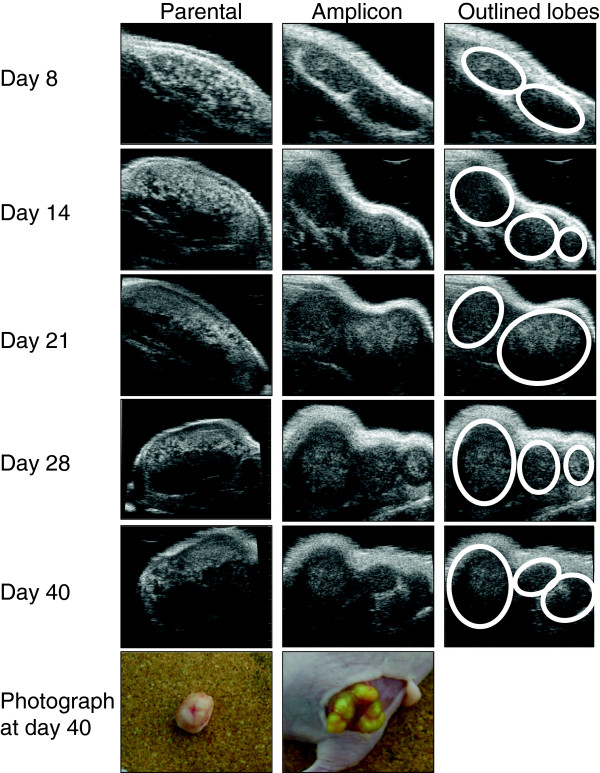
**High-frequency ultrasound imaging showing anatomical detail of xenograft growth that corresponds with *ex vivo *examination.** HF-US images of representative xenografts at the indicated day of growth. The first column shows images from an uninfected xenograft (parental cell line). Note that by day 28 the imaging plane was changed to be able to fit the xenograft into the field of view and scan over the whole tumour to generate the 3D image. The second column shows the amplicon infected xenograft. The third column outlines the lobes visible in the amplicon xenograft. The photographs in the column are of each xenograft at day 40 showing the distinct lobes of the amplicon infected xenograft compared to the uninfected xenograft.

**Figure 6:**
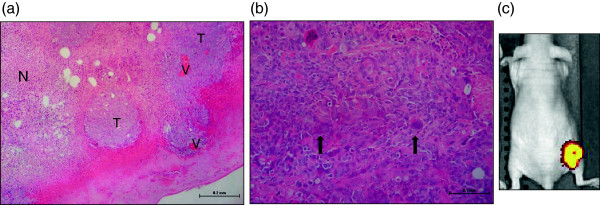
**Histological examination of the tumours.** Haematoxylin and Eosin stained sections of xenografts. **(a)** Parental cell xenografts typically showed islands of viable tumour (T) containing blood vessels (V) with large areas of necrosis (N). **(b)** amplicon infected xenografts show syncitia (arrowed) present amongst the tumour cells, which were not observed in any of the parental cell line xenografts. **(c)** The IVIS Spectrum image clearly shows the fluorescence emission from the xenograft lobes in an amplicon-infected xenograft, pseudo-coloured with the software default settings of red to yellow for increasing intensity of signal (parental cell line xenografts contained no GFP and showed no signal by BFI).

In addition to HF-US, the use of BFI allowed the persistence and expression of the amplicon to be tracked *in vivo*. The HF-US images and photographs show that the amplicon-containing xenografts grew in distinct lobes unlike the parental cell xenografts. These distinct lobes were visible even from day 8 on the HF-US images in comparison to the parental cell xenografts, thus allowing very early detection of anatomic differences between the two groups *in vivo* which was not possible to elucidate from calliper measurements alone. The detailed greyscale anatomical images using HF-US showed both lighter and darker areas (derived from areas that are more or less echogenic to ultrasound) (Figure 5). The relatively lighter areas within the xenograft were not adipose tissue and corresponded to denser tumour tissue and from histology we observed that the darker areas are necrotic tissue and when the tumours were excised open, a liquid interior core was found (Figure 6a). Amplicon infection of the cells caused formation of syncitia (fused cells) during xenograft growth, which was not evident in the parental cell xenografts, as shown in Figure 6b. The presence of lobes seen by HF-US can also be discerned in the fluorescent image taken by the IVIS Spectrum instrument (Figure 6c).

### Correlation of total radiant efficiency (fluorescence) and tumour volume measurements

**Figure 7:**
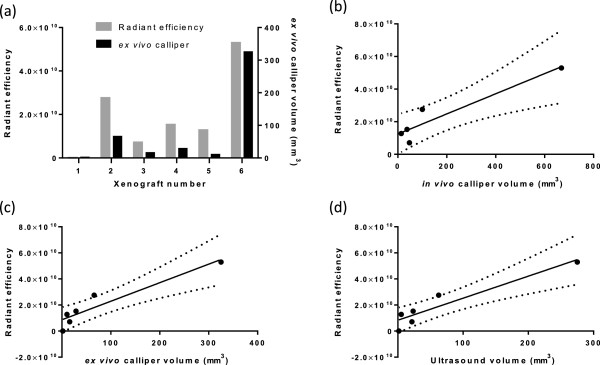
**Total radiant efficiency correlates with xenograft tumour volume. (a)** The total radiant efficiency of each amplicon-infected xenografts is plotted on the left y-axis of the graph alongside the *ex vivo* calliper tumour volume which is plotted on the right y-axis of the graph. Total radiant efficiency compared to *in vivo* calliper volume is shown **(b)**, R^2^ = 0.8882, 95% CI = 0.3568-0.9963, p = 0.0164 (n = 5 as one xenograft was too small to be measured by callipers *in vivo*). Total radiant efficiency compared to *ex vivo* calliper volumes is shown in **(c)**, R^2^ = 0.9417 95% CI = 0.5518-0.9938, p = 0.0050, n = 6. Total radiant efficiency compared to HF-US volumes is shown in **(d)**, R^2^ = 0.8895, 95% CI = 0.5606-0.9939, p = 0.0048, n = 6.

The measurement of levels of fluorescence was determined for the amplicon-infected xenografts using an IVIS Spectrum and the Living Image software and plotted alongside the *ex vivo* calliper volume (Figure 7a). These measurements show a similar pattern for the amplicon cell line in terms of fluorescence emission and calliper-derived tumour volume. *In vivo* calliper measurements on the final day of growth were less significantly correlated to fluorescence measurements than calliper measurements of the *ex vivo* xenografts (*in vivo* callipers, R^2^ = 0.8882, 95% CI = 0.3568-0.9963, p = 0.0164 compared with *ex vivo* callipers, R^2^ = 0.9417 95% CI = 0.5518-0.9938, p = 0.0050) (Figure 7b and c). HF-US volume measurements had a better correlation coefficient to fluorescence measurements than the *ex vivo* calliper measurements (R^2^ = 0.8895, 95% CI = 0.5606-0.9939, p = 0.0048) (Figure 7d). However, it must be noted these are based on small numbers in each group, as only the amplicon-infected cells contained GFP and not the parental cells.

## Discussion

Multimodal imaging in gene therapy applications is a useful tool to shed light on the behaviour of vectors during *in vivo* testing. In this study, the use of HF-US imaging identified anatomical differences during growth between the parental cell line and the vector-transfected cell line in a xenograft model, even from day 8 after implantation. It has been shown that HF-US can more accurately measure tumour volume compared to the traditional mechanical callipers, as demonstrated in this paper and by others [2,18]. The use of different ellipsoid volume formulae to generate the tumour volumes from calliper measurements made small differences in accuracy where the highest correlation to mass was found using π/6 × (L × W)^3/2^ rather than the more commonly used 0.5 × L × W^2^ as described previously (although based on only one paper [17]). Correlation to determining volume by water displacement would be the gold standard and would be a useful addition to this study. HF-US volume generation and mechanical calliper measurements by multiple operators would also be valuable for determining variability as these measurements are subject to bias from operators. Jensen and colleagues compared volumes determined by microCT, ^18^F-FDG-microPET and external callipers, to an *ex vivo* reference volume calculated by weight and density [19]. They demonstrated that micro-CT was more accurate and reproducible between observers than either external callipers or ^18^F-FDG-microPET. They also showed that ^18^F-FDG-microPET was not so useful for determining tumour size, although there was some correlation (R^2^ = 0.75). This was similar to our findings with biofluorescence imaging. As with our study, this functional tumour imaging modality is useful for metabolic imaging and should give an indication of the effect of a gene therapy vector on tumour viability. In the current study, HF-US accurately showed the slower tumour growth of the vector-transfected cell line compared to the parental cell line, as predicted from *in vitro* cell growth curves [16]. However, lobe formation was unexpected. We are currently investigating whether this is due to the GFP gene or other components of the vector backbone. We also demonstrated the utility of the different greyscale textures in monitoring different patterns of growth. The discrimination of areas of necrosis and high vascularity (using contrast agents) was also possible. This should allow real-time monitoring of agents that currently have little apparent effect on tumour volume but may have useful effects of anti-angiogenesis or inducing cell senescence. HF-US would be of particular use for very small xenografts, orthotopic models to in transgenic mice such as the *Apc*^*Min/+*^ mouse, where callipers cannot access the tumour. Indeed, gene therapy vectors are also used in non-cancer applications such as diabetes or organ regeneration, where callipers may not be used to measure disease progress or regression. In these cases, HF-US would be invaluable in monitoring progress longitudinally without sacrifice of mice.

In addition to HF-US images, the use of biofluorescence allowed monitoring of tumour growth patterns and correlated well with final tumour volumes (although it must be noted this was based on small numbers with a wide variation). This technique is a simple and very quick method of visualising the tumour and much less expensive than ^18^F-FDG-microPET, for example. Bio-fluorescence is also applicable to patients. It is currently being trialled in surgery on human tumours to define tumour margins for resection [20]. The monitoring of these two cell lines grown as xenografts showed that the presence and expression of the vector was maintained within the tumour over the duration of the experiment. This information is of great value for gene therapy applications as silencing of the vector can occur, which may not be evident from growth curves or even from immunohistochemistry on *ex vivo* tumour sections for vector proteins. Linkage of the therapeutic gene of interest to a fluorescent marker gene via an IRES (internal ribosomal entry site) sequence or as a fusion protein would yield valuable information on the efficacy of expression during the time course of an *in vivo* experiment. It may also be used to reduce costs by eliminating animals in which the introduction of a vector by injection has not been successful.

## Conclusions

In conclusion we believe that multi-modal imaging provides useful and enhanced insights into the behaviour of gene therapy vectors *in vivo*. Addition of imaging to gene therapy protocols would be straightforward especially in the case of relatively inexpensive ultrasound and biofluorescence imaging. The use of multi-modal imaging can give important information on the behaviour of gene therapy vectors in real-time, rather than traditional calliper measurements and final histological examination.

## Abbreviations

HF-US: High-frequency ultrasound; BFI: Biofluorescent imaging; HVS: Herpesvirus saimiri; GFP: Green fluorescent protein

## Competing interests

The authors declare no competing interests financial or otherwise.

## Authors’ contributions

NI carried out the experiments, analysed the data and wrote the manuscript. SAM generated the stably-infected cell line. GM imaged xenografts by HF-US and generated tumour volumes. IMC generated the amplicon. NS provided histological information on the resulting xenografts. AFM provided discussion of the results. AW was involved in study design, discussion of results and generation of the amplicon. PLC was involved in study design, discussion of results and manuscript editing. All authors read and approved the final manuscript.

## Pre-publication history

The pre-publication history for this paper can be accessed here:

http://www.biomedcentral.com/1471-2342/13/35/prepub
